# Metformin activates Wnt/β-catenin for the treatment of diabetic osteoporosis

**DOI:** 10.1186/s12902-022-01103-6

**Published:** 2022-07-22

**Authors:** Xiaopeng Huang, Siyun Li, Wenjie Lu, Longjiang Xiong

**Affiliations:** Department of Orthopedics, Jiangxi Province Hospital of Integrated Chinese & Western Medicine, Nanchang, 330003 China

**Keywords:** Diabetes mellitus (DM), Osteoporosis, Metformin, Wnt/β-catenin

## Abstract

**Background:**

With the deepening of social aging, the incidence rate of osteoporosis and diabetes continues to rise. More and more clinical studies show that diabetes is highly correlated with osteoporosis. Diabetes osteoporosis is considered as a metabolic bone disease of diabetes patients. This study aims to explore the role and mechanism of metformin (Met) in diabetic osteoporosis.

**Methods:**

Mouse MC3T3-E1 cells were treated with Met (0.5 mM) and exposed to high glucose (HG, 35 mM). The cells were cultured in an osteogenic medium for osteogenic differentiation, and the cell proliferation ability was determined using Cell Counting Kit-8; Alkaline phosphatase (ALP) activity detection and alizarin red staining were utilized to evaluate the effect of Met on MC3T3-E1 osteogenic differentiation. Western blot was used to detect the expressions of osteogenesis-related proteins (Runx2 and OCN) as well as Wnt/β-catenin signaling pathway-related proteins in MC3T3-E1 cells.

**Results:**

HG inhibited proliferation and calcification of MC3T3-E1 cells, down-regulated ALP activity, and the expression of Runx2 and OCN in MC3T3-E1 cells. Meanwhile, the activity of the Wnt/β-catenin signaling pathway was inhibited. Met treatment was found to significantly stimulate the proliferation and calcification of MC3T3-E1 cells under HG conditions, as well as increase the ALP activity and the protein expression level of Runx2 and OCN in the cells. As a result, osteogenic differentiation was promoted and osteoporosis was alleviated. Apart from this, Met also increased the protein expression level of Wnt1, β-catenin, and C-myc to activate the Wnt/β-catenin signaling pathway.

**Conclusion:**

Met can stimulate the proliferation and osteogenic differentiation of MC3T3-E1 cells under HG conditions. Met may also treat diabetic osteoporosis through Wnt/β-catenin activation.

## Introduction

Diabetes mellitus (DM) is a metabolic disease characterized by hyperglycemia and is caused by insulin resistance or inadequate insulin secretion [1]. DM is also associated with a wide range of comorbidities, such as neuropathy, nephropathy, and retinopathy. The incidence of osteoporotic fractures in patients with DM is significantly higher than in the healthy group, according to epidemiological studies [2]. The common feature of Type I DM (T1D) and type II DM (T2D) lies in the low rate of bone turnover, especially in the rate of bone formation. In comparison to those without DM, patients with T1D have a six-fold increased risk of hip fracture. Furthermore, lower bone mineral density in patients with T1D may be due to the aforementioned condition [3]. Despite having an elevated bone mineral density, patients with T2D have a 40–70% higher risk of hip fracture than patients with T1D. The reason may be attributed to the excessive insulin level in patients with T2D, which leads to collagen glycosylation and decreases bone biomechanical capacity [4]. DM patients will have hyperglycemia, which will inhibit osteoblast differentiation, reduce bone matrix synthesis, and change the regulation of parathyroid hormone on calcium and phosphorus metabolism, resulting in the decrease of calcium absorption rate and the decrease of calcium content in bones [5]. Osteoporosis may be related to hyperglycemia, hyperinsulinemia, accumulation of advanced glycation endproducts (AGEs) in collagen, decrease of serum level, increase of urinary calcium, renal failure, microangiopathy, inflammatory state, etc. in DM patients, the tendency to fall and complications are also related to the increased risk of fracture [6]. Simultaneously, studies have found a higher incidence of osteoporotic fractures in patients with DM treated with insulin [7]. The relationship between DM and osteoporosis is extremely complex, and the current mechanism of DM-induced osteoporosis remains to be elucidated. However, Manavalan et al. pointed out that because of the low rate of bone formation in patients with T1D and T2D, traditional methods of treating osteoporosis, such as inhibiting bone turnover, may not be effective in preventing bone loss and fractures in patients with T1D and T2D [8]. This led to unsatisfactory clinical treatment effects for diabetic osteoporosis. As a result, new therapeutic drugs are urgently needed to improve efficacy and thus solve the problem of diabetic osteoporosis.

Metformin (Met), a hypoglycemic drug for the first-line treatment of T2D, has a high level of safety and efficacy [9]. Studies have shown that Met plays an important role in various diseases such as cancer (breast cancer, endometrial cancer, bone cancer, colorectal cancer, and melanoma), obesity, liver disease, cardiovascular disease, and kidney disease, as well as aging [10, 11]. Meanwhile, studies have found that Met is related to cell and tissue damage as well as the risk of complications in DM. Batandier et al. [12] showed that Met inhibited hepatic gluconeogenesis in both adenosine monophosphate (AMP)-activated protein kinase (AMPK)-dependent and independent manner, and that it inhibited hepatic gluconeogenesis and enhanced insulin sensitivity by entering hepatocytes via organic cation transporter 1 to inhibit mitochondrial respiratory complex I and cause a decrease in adenosine-triphosphate (ATP) synthesis. Other studies have found an association between the application of Met and a reduced risk of microvascular and macrovascular complications in patients with T2D [13]. Simultaneously, Zhai et al. discovered that Met improved podocyte injury by restoring the expression of nephrin in the kidney tissue of T2D rats [14]. Furthermore, Kim et al. suggested that the inhibitory effect of Met on oxidative damage could inhibit podocyte loss in diabetic nephropathy, thereby protecting renal function [15]. Overall, Met may be useful in the treatment of DM and its corresponding complications. Recent studies suggest that AMPK is an important mediator of homeostasis. It is involved not only in glucose metabolism, but also in osteogenesis. AMPK can directly affect the formation of mature and high-quality bone by reducing osteoclasts, increasing the formation of osteoblasts and enhancing bone mineral deposition [16]. As an activator of AMPK, we questioned whether Met could be used as a therapeutic drug for diabetic osteoporosis because of its regulating effect on energy metabolism and cell proliferation. As a result, MC3T3 cells were used in this study to evaluate the effects of Met on embryonic osteoblast proliferation, calcification, and osteogenic differentiation in the high glucose (HG) environment. Furthermore, we preliminarily explored whether Met alleviated the adverse effects of the HG environment on cells through the Wnt/β-catenin signaling pathway. The efficacy of Met in treating DM osteoporosis was also evaluated.

## Material and methods

### Cell culture and treatment

The mouse embryonic osteoblast cell line MC3T3-E1 was purchased from the Cell Bank of the Chinese Academy of Sciences. MC3T3-E1 was cultured in α-modified minimal essential medium (α-MEM, Hyclone, USA) containing 10% fetal bovine serum (Gibco, USA), 100 × penicillin-streptomycin, and 25 μg/mL amphotericin. After that, the medium was cultured at 37 °C with 5% CO_2_ in an incubator. Then, in a six-well plate, MC3T3-E1 cells were inoculated at 1 × 10^4^ cells/well for subsequent culture in an incubator. After the cells grew to 80% confluence, they were added into an osteogenic medium (50 μg/ml ascorbic acid, 100 mM dexamethasone, and 10 mM β-glycerophosphate).

The cells were grouped into four and treated as follows. In the control group, MC3T3-E1 cells did not receive any treatment; in the Met group, MC3T3-E1 cells were treated with 0.5 mM metformin (Met) [17] for 48 h; in the HG group, MC3T3-E1 cells were treated with 35 mM glucose for 48 h; in the HG + Met group, MC3T3-E1 cells were first treated with 35 mM glucose for 48 h, followed by 0.5 mM Met treatment for 48 h.

### Cell counting Kit-8 (CCK-8)

CCK-8 assay was used to assess cell proliferation. Specifically, 100 μL of MC3T3-E1 cells were seeded at 1 × 10^4^ cells/well in a 96-well plate, and different treatments were performed after 24 hours. After 24 hours of treatment, a cell proliferation assay was performed according to the instructions of the CCK-8 kit (MCE, USA). 10 μL of CCK-8 solution was added to the wells for 2-h incubation at 37 °C to make it clear. Finally, an enzyme-labeled instrument was used to measure the absorbance at 450 nm. Each group was set up with six duplicate wells, and all experiments were repeated at least three times.

### Alkaline phosphatase (ALP) activity assay

The osteogenic differentiation-induced MC3T3-E1 cells in each group were taken, and the total protein was extracted using radio immune precipitation assay (RIPA) cell lysate (Solebo, China) on ice. The samples were then centrifuged for 15 min at 4 °C and 10,000 rpm. The total protein concentration was determined using a Bicinchoninic acid (BCA) kit (Beyotime, China). The absorbance at a wavelength of 520 nm was detected using an ultraviolet-visible spectrophotometer, and the ALP activity was evaluated according to the instructions of the ALP colorimetric test kit (Elabscience, China).

### Alizarin red staining (ARS)

To assess cell calcification, 14-d cultured MC3T3-E1 cells were stained with alizarin red. The cells were fixed with 4% paraformaldehyde for 20 min and then stained for 15 min according to the instructions of the osteoblast mineralization nodule staining kit (Beyotime, China). The staining solution was discarded and the stained cells were rinsed when red particles appeared. The washed cells were then examined and photographed under a microscope, with untreated cells serving as a Control group.

### Western blot

The total protein of the cells to be tested was extracted using RIPA cell lysate (Solebo, China) on ice, and then subjected to cryogenic sonication. The supernatant was taken as total protein after centrifugation at 10,000 rpm for 20 min at 4 °C. The concentration of the extracted protein was determined using a BCA kit (Beyotime, China). Denaturation was achieved by boiling 20 μg of protein supplement with 5 × loading buffer. Sodium dodecyl sulfate-polyacrylamide gel electrophoresis (SDS-PAGE) was then used to separate the protein. The protein was then transferred to the polyvinylidene fluoride (PVDF) membrane after SDS-PAGE electrophoresis. After that, the membrane was blocked using a blocking solution containing 5% nonfat dry milk for 1–2 h. After washing with tris buffered saline buffer with tween 20 (TBST), the membrane was incubated with primary antibodies (Runx2, ab76956, 1:1000; OCN, ab93876, 1:1000; Wnt1, ab15251, 1:1000; β-catenin, ab32572, 1:2000; c-myc, ab32072, 1:1000; β-actin, ab8226, 1:5000; all from Abcam, USA) overnight at 4 °C. The membranes were then rinsed three times with TBST, a horseradish peroxidase (HRP)-conjugated secondary antibody (Abcam, USA, 1:5000) was added for incubation at 25 °C for 1 hour. Finally, the membrane was rinsed with TBST three times. The protein was then developed in a gel imaging system using enhanced chemiluminescence (ECL) reagent (Beyotime, China), and images were taken. The gray level of the protein bands was analyzed using Image J software, and β-actin was utilized as an internal reference for calculating relative protein expression.

### Statistics

All analyzed data were expressed as mean ± standard deviation (SD) and graphed by Graphpad prism 9. SPSS 22.0 software was performed for the statistical analysis. The Shapiro Wilk (S-W) test was used to evaluate whether the data were normally distributed, and a one-way analysis of variance was utilized to assess the significance of differences between groups. Furthermore, *p* < 0.05 was regarded as the judging criterion of significant difference.

## Results

### Metformin promotes the proliferation of MC3T3-E1 cells under HG condition

**Fig. 1:**
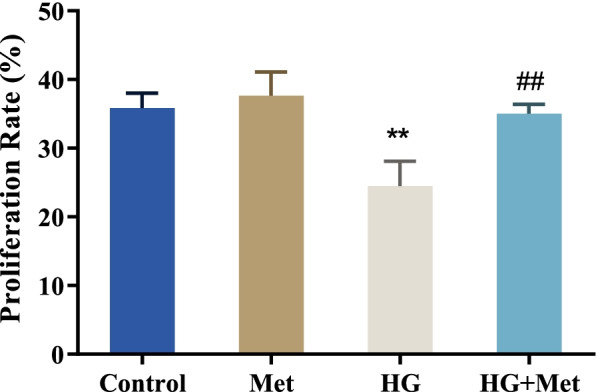
Metformin promotes the proliferation of MC3T3-E1 cells under HG conditions. CCK-8 was applied to detect the proliferation of cells in each group, **P < 0.01, vs. Control; ##*P* < 0.01, vs. HG. Met, metformin; HG, 35 mM glucose

The HG environment was created in vitro to better understand the influence of Met on the formation of diabetic osteoblasts. The effect of Met on the proliferation of MC3T3-E1 cells in an HG environment was then assessed using a CCK-8 assay. The results showed that 0.5 mM Met had no effect on MC3T3-E1 cell growth, whereas HG (35 mM glucose treatment) remarkably inhibited MC3T3-E1 cell proliferation (*p* < 0.01). Furthermore, 0.5 mM Met treatment could significantly increase the proliferation of MC3T3-E1 cells in the HG group (*p* < 0.01) (Fig. 1), suggesting that Met could promote the proliferation of MC3T3-E1 cells under HG conditions.

### Metformin enhances the osteogenic differentiation ability of MC3T3-E1 cells under HG condition

**Fig. 2:**
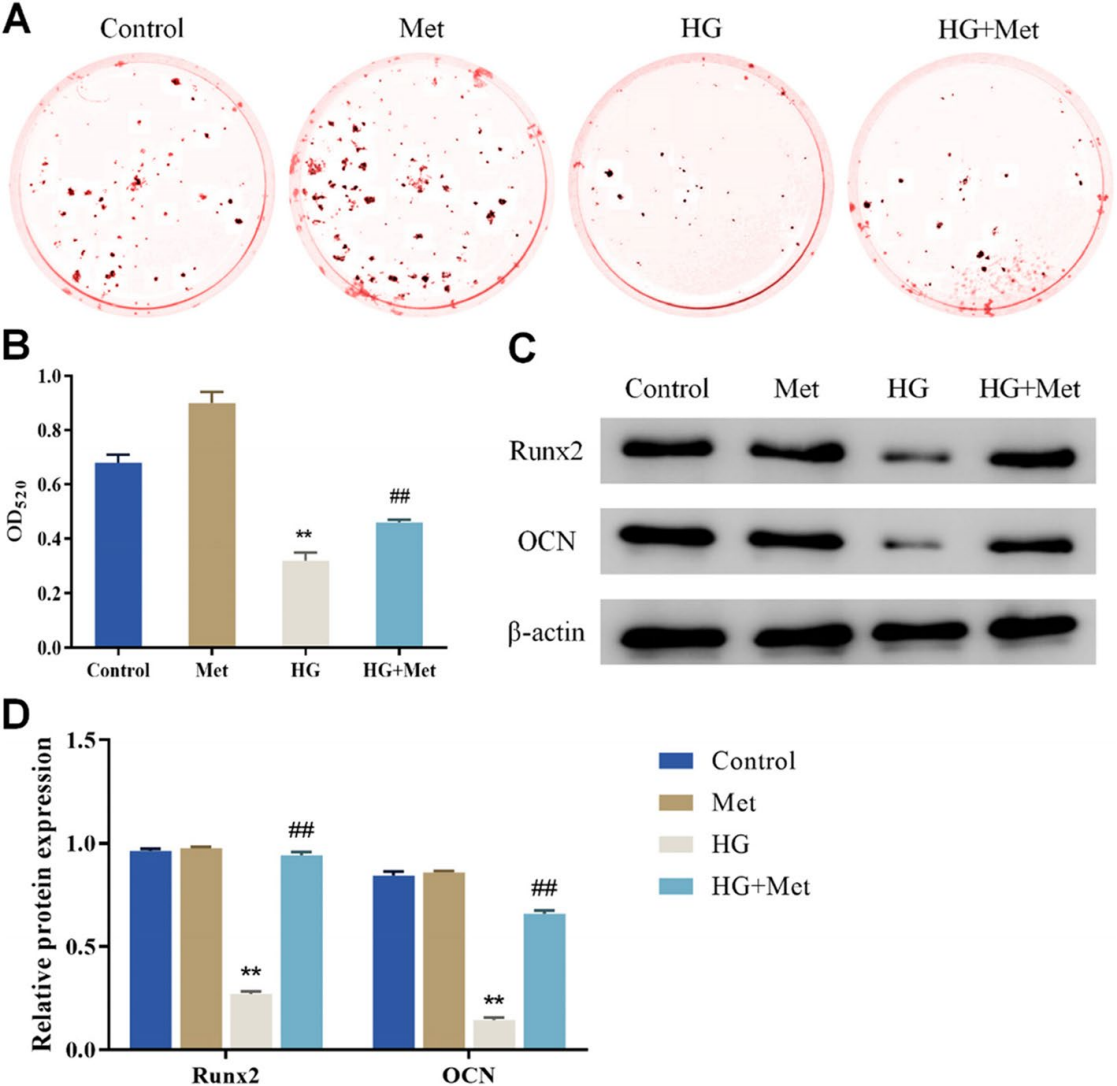
Metformin enhances the osteogenic differentiation ability of MC3T3-E1 cells under HG conditions. **A** ARS was applied for the assessment of calcification in MC3T3-E1 cells in each group. **B** ALP colorimetric assay kit was utilized to measure the activity of ALP in each group of cells; **C**/**D** Western blot was performed for the detection of the expression of osteogenic differentiation-related genes (Runx2, OCN) in each group of cells, **P < 0.01, vs. Control; ##*P* < 0.01, vs. HG

ALP, Runx2, and OCN are markers of osteoblast differentiation. As a result, the protein expression level of ALP, Runx2, and OCN was detected to explore the effect of Met on the osteogenic differentiation of MC3T3-E1 cells under HG conditions. To explore the role of Met in the ability of MC3T3-E1 cells to form mineralized extracellular matrix under HG conditions, the formation of mineralized extracellular matrix was also studied in the cells. The results of ARS revealed that compared with the Control group, the ARS in the cells of the HG group was lighter; while compared with the HG group, the ARS in the cells of the HG + Met group was observably increased (Fig. 2A). Meanwhile, HG could significantly reduce the viability of ALP in MC3T3-E1 cells but Met dramatically increased the ALP viability of MC3T3-E1 cells in the HG group (Fig. 2B). Furthermore, western blot results revealed that the protein expression level of Runx2 and OCN in the cells of the HG group was decreased when compared to the Control group; however, the protein expression level of Runx2 and OCN in the cells of the HG + Met group was observably increased when compared to the HG group (Fig. 2C/D). According to the findings, HG treatment led to the decrease of calcification and osteogenic differentiation ability of MC3T3-E1 cells, whereas Met dramatically increased the calcification and osteogenic differentiation of MC3T3-E1 cells.

### Metformin activates the Wnt/β-catenin pathway in MC3T3-E1 cells under HG condition

**Fig. 3:**
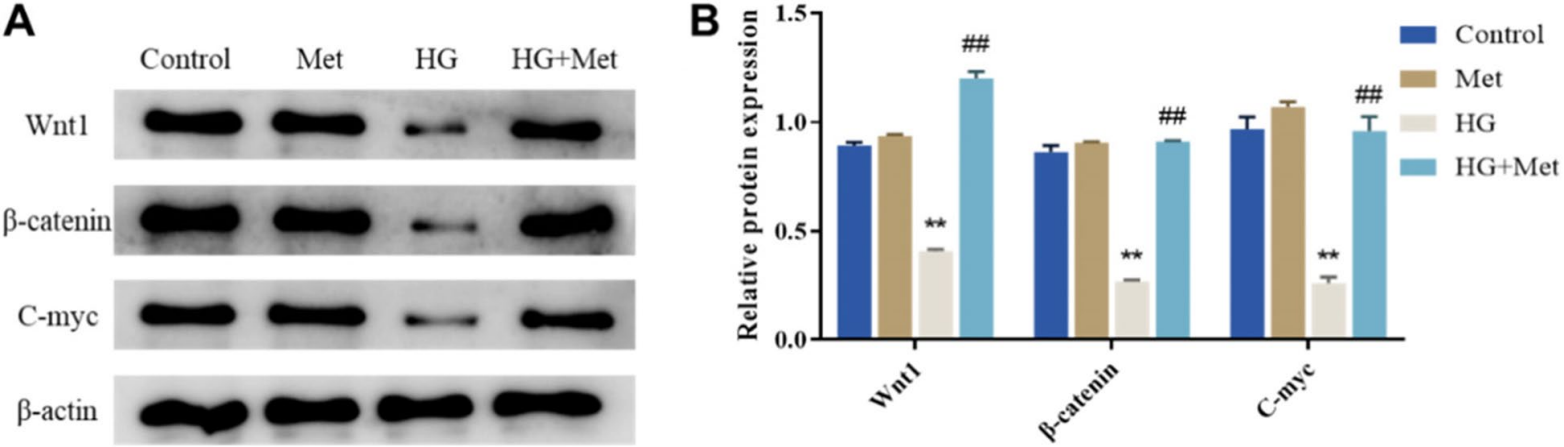
Metformin activates the Wnt/β-catenin pathway in MC3T3-E1 cells under HG conditions. **A**/**B** Western blot to detect the expression of Wnt/β-catenin pathway-related proteins (Wnt1, β-catenin, and C-myc) in cells of each group, **P < 0.01, vs. Control; ##*P* < 0.01, vs. HG

Studies have disclosed that the Wnt/β-catenin signaling pathway plays an important role in the proliferation and osteogenic differentiation of MC3T3-E1 cells [18]. Western blot was applied to detect the expression level of Wnt/β-catenin signaling pathway-related proteins in each group of cells to clarify whether Met regulated Wnt/β-catenin to promote proliferation and osteogenic differentiation of MC3T3-E1 cells. As a result, the protein expression level of Wnt1, β-catenin, and C-myc in the cells of the HG group was lower than that in the Control group; whereas the protein expression level of Wnt1, β-catenin, and C-myc in the cells of the HG + Met group were noticeably greater than those in the HG group (Fig. 3A/B). The above indicated that HG treatment inhibited the activity of the Wnt/β-catenin pathway in MC3T3-E1 cells, while Met could activate the Wnt/β-catenin pathway in MC3T3-E1 cells under HG conditions.

## Discussion

DM affects bone by impairing glucose metabolism, disrupting skeletal microvascular function and muscle endocrine function, as well as producing oxidative derivatives. Furthermore, chronic hyperglycemia regulates osteoblast gene expression, function, and bone formation, thereby leading to bone loss [19]. In patients with DM, advanced glycation end products were accumulated and lower bone turnover was observed in collagen. As a result, in patients with DM, bone strength and fracture risk may be decreased and increased respectively [20]. Furthermore, because of the strong heterogeneity of patients with DM, it was difficult to find specific markers of metabolic disorders or risk regulators. As a result, precisely targeted therapy was difficult to adopt in clinical practice. Nonetheless, as the main characteristic of DM, the rate of bone turnover is slowed. This demonstrated that osteoblasts could be influenced directly or indirectly [21]. In this study, the HG environment inhibited the proliferation, calcification, and osteogenic differentiation of MC3T3-E1 cells. Yang et al. also found that the activity and calcification of osteogenic MC3T3-E1 cells were remarkably decreased after HG stimulation. After prolonged HG treatment, the MC3T3-E1 cells were more severely damaged [1]. We hypothesized that the HG-induced MC3T3-E1 cells growth inhibition was due to enhanced expression of PPARγ induced by HG [22], which inhibited cell proliferation and promoted the formation of adipocytes [23]. On the other hand, Met significantly increased the proliferation level of MC3T3-E1. Different methods of Met in inhibiting adipogenesis may be served as an explanation for the above situation. On one hand, the inhibitory effect of Met on adipogenesis could be realized through AMPK activation and PPARγ downregulation; on the other hand, the effect could also be achieved by an AMPK-independent mechanism [24]. Met, for example, could regulate the ROS-AKT-mTOR axis to increase the proliferation and osteogenic differentiation of mesenchymal stem cells (MSCs) under HG conditions, according to Zhou et al. [25]. Interestingly, Met often inhibits cell proliferation in cancer, while promoting the proliferation of osteoblasts or MSCs in an HG environment. This reflected the great medical value of the Met.

Met has been shown to play a role in osteogenic differentiation, primarily in the maintenance of bone density, bone loss antagonization, and bone microstructure protection [26]. This study also revealed that Met significantly increased the ALP activity of cells and stimulated the expression of osteogenesis-related proteins (Runx2 and OCN). Furthermore, Gu et al. demonstrated the role of Met in stimulating the osteogenic differentiation of MSCs [27]. Met has been shown to be an activator of the AMPK signaling pathway in previous studies, with AMPK signaling activation subsequently stimulating bone formation and increasing bone mass in bone physiology [28]. However, additional studies have found that AMPK activity decreases over time during osteoblast differentiation. This is because a large amount of energy is required for bone matrix production and mineralization during differentiation [29]. As a result, we speculated that Met activated another signaling pathway to ensure the cell regulation throughout the whole differentiation process. The Wnt/β-catenin pathway, also known as the canonical Wnt pathway, is not only involved in the formation of DM but also is the key to osteoporosis [30]. Recent studies have found that the Wnt/β-catenin pathway is closely related to bone formation [31]. To make it clear, Wnt proteins act on cell surface receptors to prevent β-catenin degradation. The accumulating β-catenin translocates into the nucleus sequentially, stimulating the expression of various downstream genes [32, 33]. Multiple studies have shown that the expression of Wnt6, Wnt10a, and Wnt10b inhibits the differentiation of MSCs into adipocytes while promoting MSCs differentiation into osteoblasts through the canonical Wnt pathway [34]. Furthermore, lots of studies have revealed that Wnt/β-catenin plays an important role in the proliferation and differentiation of embryonic osteoblasts [35, 36]. Met also activated the Wnt/β-catenin signaling pathway to reduce inflammation and apoptosis, according to Zhang et al. [37]. Based on this, we speculated that Met exerted effects on promoting proliferation and osteogenic differentiation in embryonic osteoblasts through the Wnt/β-catenin signaling pathway. Furthermore, the western blot further confirmed that the activity of the Wnt/β-catenin signaling pathway was significantly decreased in the HG environment. Met appeared to be a potential activator of the Wnt/β-catenin signaling pathway, based on the information presented above.

However, this study still has limitations. First, because the mechanism of action was not verified by pathway activators or inhibitors in this study, the direct relationship could not be determined. In addition, the anti-osteoporosis effect in HG cells of Met was only explored in vitro experiments, while the situation in vivo remained uncertain. As a result, a significant number of basic experiments are required to explore its role and specific mechanism, to provide more comprehensive experimental data support for the wide application of Met. For example, experiments can be conducted on animal models of diabetes, and patient samples can also be collected for comparison in clinical research.

## Conclusion

In conclusion, Met can promote the proliferation and osteogenic differentiation of MC3T3-E1 cells and also alleviate osteoporosis. Further exploration disclosed that the above effects of Met may be associated with its activation of the Wnt/β-catenin signaling pathway. In addition, this study provides new drug therapy for the treatment of diabetic osteoporosis.

## Data Availability

The datasets used and/or analysed during the current study are available from the corresponding author on reasonable request.

## Abbreviations

Met: metformin
ALP: Alkaline phosphatase
DM: Diabetes mellitus
AMP: Adenosine monophosphate
AMPK: Activated protein kinase
HG: High glucose
ECL: Enhanced chemiluminescence
SD: Standard deviation
